# 3D-printed living implants: advancing cranial reconstruction

**DOI:** 10.1097/MS9.0000000000004248

**Published:** 2025-10-31

**Authors:** Muhammad Khizar, Muhammad Zaib, Muhammad Bilal, Hasiba Karimi, Hasibullah Aminpoor

**Affiliations:** a Faculty of Medicine, Georgian American University, Manifest Medical Research Co, Tbilisi, Georgia; b Faculty of Medicine, Shahida Islam Medical and Dental College, Lodhran, Punjab, Pakistan; c Faculty of Medicine, Bezmialem Vakif University, Istanbul, Turkey; d Faculty of Medicine, Kabul University of Medical Sciences “Abu Ali Ibn Sina”, Kabul, Afghanistan

**Keywords:** 3D printing, biodegradable scaffolds, cranial reconstruction, neurosurgery, regenerative medicine, tissue engineering

## Abstract

Severe cranial defects caused by trauma, tumor resection, or congenital anomalies remain challenging to repair. Conventional cranioplasty using autografts or metal/polymer plates carries risks of donor-site morbidity, infection, and suboptimal contour. Patient-specific 3D-printed “living” implant, biodegradable scaffolds seeded with autologous cells, offer precise anatomical fit and active bone regeneration. Early clinical applications in countries including South Korea, Egypt, and Brazil demonstrate improved cosmetic outcomes, accelerated bone formation, and reduced operative time. Integrating tissue engineering with additive manufacturing represents a shift in neurosurgical reconstruction, providing biologically active solutions that restore both form and function. Ongoing research focuses on vascularization, cell viability, and clinical translation. Living 3D-printed cranial implants have the potential to redefine standards of care, emphasizing regenerative repair over inert prosthetic replacement.

*To the Editor,*

Cranial defects following trauma, decompressive craniectomy, tumor excision, or congenital anomalies present significant reconstructive challenges. Traditional cranioplasty approaches using autologous bone grafts or prefabricated metal/polymer plates are limited by donor-site morbidity, infection risk, supply constraints, and suboptimal aesthetic outcomes^[1,2]^. Patient-specific 3D printed implants have emerged as a promising alternative, providing precise anatomical fit and enhanced functional and cosmetic results^[1]^.

High-resolution imaging combined with computer-aided design allows fabrication of customized scaffolds. Park *et al* in South Korea successfully employed polycaprolactone/β-tricalcium phosphate (PCL/β-TCP) 3D-printed implants to restore complex skull deformities, achieving smooth contours and biocompatibility^[2]^. Similarly, Elnaggar *et al* in Egypt demonstrated successful integration and durable reconstruction using patient-specific polyetheretherketone implants^[3]^. These international experiences underscore the feasibility of additive manufacturing in improving surgical outcomes and patient satisfaction^[1,3]^.

Moving beyond inert implants, biologically active scaffolds integrate tissue engineering strategies, seeding 3D-printed matrices with “autologous osteogenic cells or growth factors” to promote in situ bone regeneration. In Brazil, Pitol-Palin *et al* reported that stem cell-seeded βTCP scaffolds significantly accelerated calvarial bone formation compared to scaffolds alone^[4]^. Recent reviews highlight the potential of 3D bioprinting for precise craniofacial tissue engineering, enabling implants that not only restore contour but also actively regenerate bone^[5]^. Our work is in line with the TITAN Guidelines on the need for transparency in AI use in healthcare^[6]^.

In conclusion, 3D-printed living cranial implants represent a transformative advancement in neurosurgery. Customized scaffolds improve fit, reduce operative time, and enhance outcomes, while the incorporation of living cells offers true regenerative potential. Multidisciplinary collaboration is essential for optimizing clinical translation, standardizing protocols, and ensuring safety. Continued innovation may redefine cranial reconstruction by restoring both form and function with biologically active implants.
